# Digital Pacifier for Parents: A Cross-Sectional Study of Mobile Phone Use, Patterns, and Parental Attitudes in Young Children in India

**DOI:** 10.7759/cureus.107676

**Published:** 2026-04-24

**Authors:** Arpit Suman, Jagdish Mujalda, Gayatri K Gupta, Manisha Manisha, Gururaja R, Dinesh Yadav, Omna Shaki

**Affiliations:** 1 Preventive Medicine, Shri Ram Murti Smarak Institute of Medical Sciences (SRMS IMS) Bhojipura Bareilly, Bareilly, IND; 2 Pediatrics, Military Hospital, Sagar, IND; 3 Dermatology, Skin Diseases Centre, Nashik, Nashik, IND; 4 Ophthalmology, Seth Sooraj Mal Jatia (SSMJ) Government Hospital, Khurja, IND; 5 Pediatrics, Military Hospital, Jodhpur, IND; 6 Pediatrics, Military Hospital, Jaipur, IND; 7 Trauma and Emergency, 306 Field Hospital, Bareilly, IND

**Keywords:** digital pacifier, early childhood, indian parenting practices, mobile phone, screen time

## Abstract

Background

The increasing reliance on smartphones and digital media to calm or occupy young children, often referred to as the “digital pacifier,” has become widespread in Indian households. While this approach provides immediate convenience to caregivers, emerging research indicates potential adverse impacts on sleep patterns, behavior, and early developmental outcomes. Sociodemographic factors are likely to significantly influence this practice.

Objective

This study aims to characterize the sociodemographic characteristics and associated factors influencing the use of digital devices as tools for calming or engagement.

Methods

A cross-sectional observational study was carried out among 320 children aged 1-5 years. Data on age, gender, family structure, parental education, employment status, socioeconomic background, and children’s screen exposure were obtained using a structured questionnaire. Descriptive statistical methods were applied, and findings were expressed as frequencies and percentages.

Results

Among 320 children, 248 (77.5%) were male and 72 (22.5%) were female. The mean daily screen exposure showed a progressive increase with age, ranging from nearly 2 hours/day in 1-year-olds to approximately 5-6 hours/day in children aged 4-5 years. Children belonging to nuclear families demonstrated greater screen use (4.5 ± 1.2 hours/day) compared to those from joint families (3.2 ± 1.0 hours/day). Children of illiterate mothers had significantly higher screen exposure compared to those of literate mothers (4.1 ± 1.2 versus 3.4 ± 1.1 hours/day; *t* = 2.94, *p* = 0.004). Similarly, children of working mothers demonstrated greater screen time than those of non-working mothers (4.6 ± 1.3 versus 3.5 ± 1.1 hours/day; *t* = 5.48, *p* = 0.002). The highest screen exposure was observed among children from dual-income families, where both parents were working (4.8 ± 1.3 versus 3.6 ± 1.1 hours/day; *t* = 6.21, *p* = 0.001). No statistically significant association was found with paternal education or employment.

Conclusion

Findings highlight the significant role of family dynamics, parental responsibilities, and socioeconomic context in shaping early childhood screen exposure. Addressing the “digital pacifier” phenomenon necessitates targeted awareness initiatives and culturally sensitive recommendations to promote healthier developmental environments for young children.

## Introduction

The rapid expansion of digital technology within Indian households has substantially altered the environment in which young children grow and develop. Smartphones, originally designed for communication and productivity, have increasingly become versatile tools used for entertainment, distraction, and behavioral management in early childhood. This emerging behavior, commonly termed “digital pacifying,” describes the practice whereby caregivers provide mobile devices to soothe, distract, or engage children during periods when attention, quietness, or time management is required [1]. This pattern is now frequently observed across diverse settings, including homes, restaurants, clinics, social gatherings, and during travel, indicating a marked shift in caregiving behavior with potential developmental consequences.

The Indian context presents unique challenges that may contribute to this trend. Factors such as high population density, limited availability of safe recreational spaces, and increasingly demanding work schedules make it difficult for caregivers to balance childcare with other responsibilities [2]. Additionally, the transition toward nuclear family structures and reduced availability of extended family support systems have further increased dependence on digital tools for managing young children [3]. Concurrently, the widespread accessibility of low-cost smartphones and readily available digital content has made screen-based engagement one of the most convenient and immediate solutions for behavioral management.

Evidence from Indian studies indicates that exposure to digital media begins at a very early age. Some infants are introduced to screens as early as 2 months, with a median age of initiation around 10 months. By 18 months of age, a majority of children have already been exposed to screen media. Notably, smartphone use (96%) has surpassed television viewing (89%) among young children in these reports [4]. Excessive screen exposure during early developmental stages has been linked to various adverse outcomes, including reduced physical activity, increased risk of obesity, sleep disturbances, and concerns related to cognitive and behavioral development [5]. While previous Indian research has examined mobile phone use among children for multiple purposes [6,7], specific aspects, such as the use of devices as “digital pacifiers” or perceived parental “saviors,” remain insufficiently explored.

The present study was designed as an exploratory assessment of screen exposure patterns and the use of mobile phones as a “digital pacifier” among young children in a real-world caregiving context. Given the limited context-specific data from Indian settings, particularly regarding routine caregiving practices and family-level influences, this study aimed to examine associations between screen exposure and selected developmental and sociodemographic factors rather than to establish causal relationships.

The present study aimed to assess screen exposure patterns among young children and to evaluate the use of mobile phones as a “digital pacifier” in routine caregiving situations. Additionally, the study sought to examine the association of screen exposure with selected developmental indicators and to explore the influence of parental and sociodemographic factors on children’s screen use.

## Materials and methods

This cross-sectional observational study was undertaken to evaluate sociodemographic characteristics and contextual determinants associated with digital device use among young children. A total of 320 families, each with one child aged 1-5 years, were included during the study period from October 2025 to January 2026 in Bareilly district, Uttar Pradesh, India.

Inclusion and exclusion criteria

Children aged 1-5 years, accompanied by parents or primary caregivers who provided written informed consent, were eligible for inclusion. Only families residing within the study area during the study period were considered.

Exclusion criteria included refusal to participate, presence of diagnosed developmental disorders or chronic neurological conditions that could independently influence behavioral patterns or screen use, and inability to provide reliable information due to unavailability or communication limitations. The study focused on a general pediatric population and was not designed as a case-control study to compare children with specific developmental disorders.

Study population

The study population comprised caregivers (parents or legal guardians) of children aged 1-5 years attending the study facility. Caregivers who were primarily responsible for the child’s daily care and were willing to participate were included in the study.

Family type definition

Family type was categorized as nuclear or joint family. A joint family was defined as a household in which extended family members, including grandparents, reside together and share caregiving responsibilities.

Study instrument

In this study, “parental perceptions” refers to caregivers’ reported attitudes, practices, and reliance on mobile phones in routine child-rearing situations. Sociodemographic factors include variables such as family structure, parental education, occupation, and caregiving environment that may influence children’s screen exposure.

Data collection

Data were collected from caregivers using a predesigned, structured questionnaire developed by the study team and administered to all participating families. The detailed questionnaire is provided in the Appendices. The tool comprised multiple domains, including use during feeding, household work, travel, public settings, to stop crying, during parental work, to keep the child engaged, and for obtaining personal time. Each domain contained three items, and responses were recorded on a four-point Likert scale (never, sometimes, often, and always). Information collected included child demographics (age and gender), family characteristics (family type and co-residence with grandparents), parental education (literate/illiterate), occupation (employed/unemployed), and socioeconomic status (classified as debt or savings). Additional details regarding caregiving practices and contextual use of digital devices as calming or engagement tools (“digital pacifier”) were also recorded.

Statistical analysis

Data entry was performed using spreadsheet software and analyzed with appropriate statistical tools (e.g., SPSS version 19, IBM Corp., Armonk, NY). Descriptive statistics were used to summarize variables. Categorical data were expressed as frequencies and percentages. Associations between variables were assessed using the chi-square test or Fisher’s exact test, as applicable. A p-value < 0.05 was considered statistically significant.

Ethical clearance was obtained from the institutional ethics committee (approval number: FD/03/2025), and all procedures adhered to established ethical standards. Written informed consent was obtained from parents.

## Results

**Figure 1:**
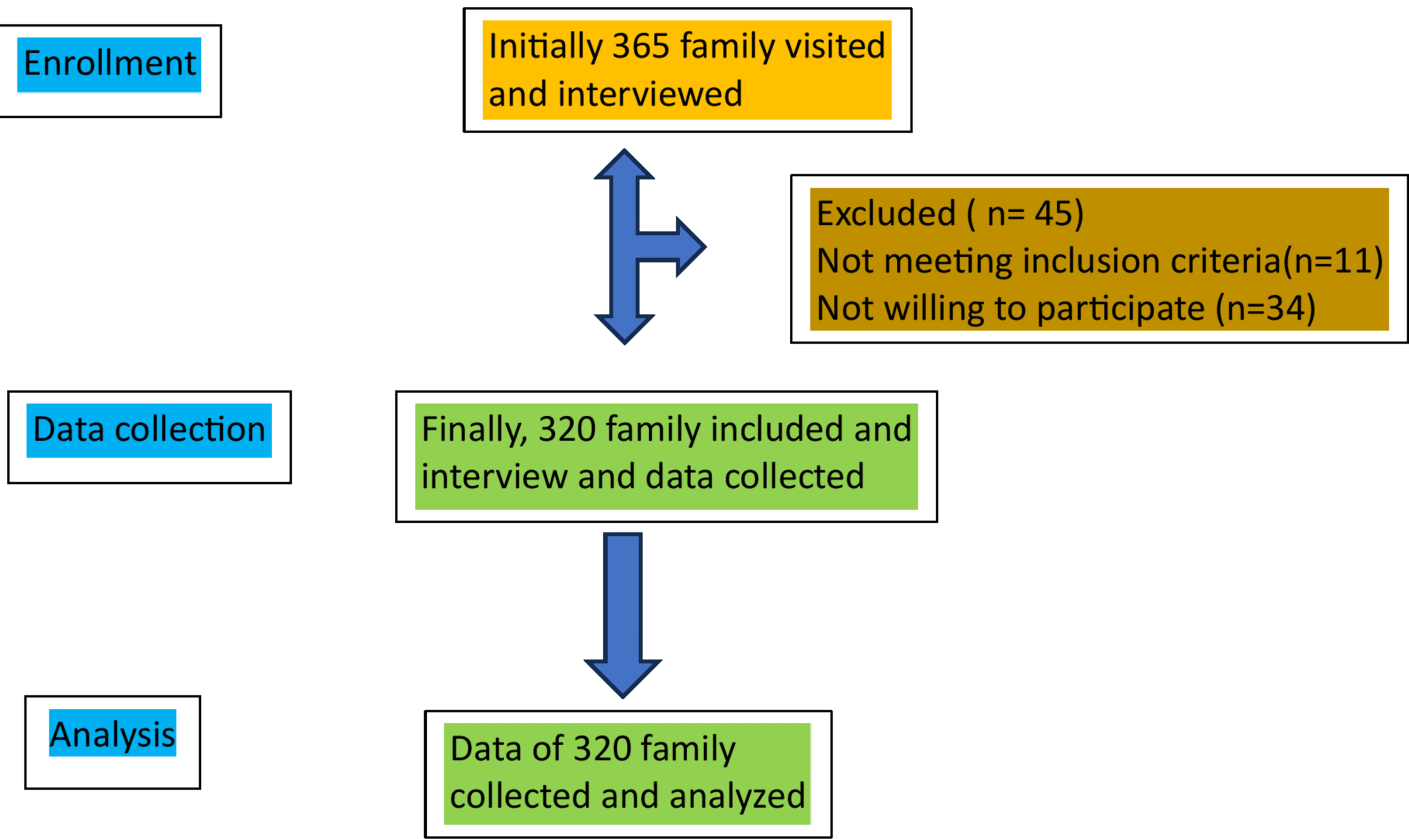
Flow diagram of participant recruitment and inclusion Note: This figure was created using the Microsoft Office tool.

A total of 365 families were initially approached and interviewed. Of these, 45 (12.3%) were excluded, including 11 (3.0%) who did not satisfy the inclusion criteria and 34 (9.3%) for other reasons. Ultimately, 320 (87.7%) families were included, and complete data collection was achieved (Figure 1).

The study population comprised 320 children, with a predominance of males (248 (77.5%)) compared to females (72 (22.5%)). The majority of participants were in the 3-5-year age group (210 (65.7%)), while 61 (19.0%) were aged 1 year. Most children belonged to nuclear families (184 (57.5%)), followed by joint families (136 (42.5%)).

Parental education showed a near-equal distribution among mothers, with 156 (48.8%) being literate and 164 (51.2%) illiterate. In comparison, a higher proportion of fathers were literate (197 (61.6%)), whereas 123 (38.4%) were illiterate.

Regarding employment status, the majority of mothers were not engaged in formal employment (234 (73.1%)), while 86 (26.9%) were employed. In contrast, most fathers were employed (306 (95.6%)), with only 14 (4.4%) being unemployed. Overall, both parents were employed in 79 (24.6%) households (Table 1).

**Table 1:** Demographic characteristics of included families (N = 320)

Variables	Number (%)
Child age (years)	
1	61 (19.0)
2	49 (15.3)
3	80 (25.0)
4	70 (21.9)
5	60 (18.8)
Gender	
Male	248 (77.5)
Female	72 (22.5)
Family type	
Nuclear	184 (57.5)
Joint	136 (42.5)
Education status of mother	
Literate	156 (48.8)
Illiterate	164 (51.2)
Education status of father	
Literate	197 (61.6)
Illiterate	123 (38.4)
Working status of mother	
Employed	86 (26.9)
Unemployed	234 (73.1)
Working status of father	
Employed	306 (95.6)
Father unemployed	14 (4.4)
Both parent working	79 (24.6)

A statistically significant rise in average daily screen time was noted with increasing age, with children in the 4-5-year age group exhibiting the highest exposure (4-6 hours/day), whereas 1-year-old children had an average exposure of approximately 2 hours/day (p < 0.001) (Table 2).

**Table 2:** Time spent on mobile by children according to age of child (N = 320) Note: Values are expressed as mean ± SD. Statistical analysis was performed using one-way ANOVA. A statistically significant increase in screen time was observed across age groups (F(4,315) = 18.72, p < 0.001). Post hoc analysis using Tukey’s test demonstrated significant differences between age groups. A p-value < 0.05 was considered statistically significant. SD: standard deviation, ANOVA: analysis of variance

Variables	Number (%)	Time spent (in hours) on mobile/day	Statistical value	p-value
Age (years)			F(4,315) = 18.72	<0.001
1	61 (19.0)	2.0 ± 0.6		
2	49 (15.3)	3.2 ± 0.8		
3	80 (25.0)	4.1 ± 1.0		
4	70 (21.9)	5.0 ± 1.1		
5	60 (18.8)	5.6 ± 1.2		

The distribution of children based on family structure and parental characteristics, along with corresponding average daily screen time, is presented in Table 3.

**Table 3:** Association of sociodemographic factors with screen time in children Note: Values are expressed as mean ± SD. Independent sample t-test was used for comparison between the two groups. A p-value < 0.05 was considered statistically significant. SD: standard deviation

Characteristics	Number (%)	Mean screen time (hours/day)	Statistical value (t)	p-value
Family type			7.82	<0.001
Nuclear	184 (57.5)	4.5 ± 1.2	-	
Joint	136 (42.5)	3.2 ± 1.0	-	
Mother’s education			2.94	0.004
Literate	156 (48.8)	3.4 ± 1.1	-	
Illiterate	164 (51.2)	4.1 ± 1.2	-	
Father’s education			1.32	0.187
Literate	197 (61.6)	3.7 ± 1.1	-	
Illiterate	123 (38.4)	4.6 ± 1.3	-	
Mother’s working status			5.48	0.002
Employed	86 (26.9)	4.6 ± 1.3	-	
Unemployed	234 (73.1)	3.5 ± 1.1	-	
Father’s working status			0.76	0.382
Employed	306 (95.6)	3.2 ± 1.2	-	
Unemployed	14 (4.4)	4.6 ± 1.0	-	
Both parents working			6.21	0.001
Yes	79 (24.6)	4.8 ± 1.3	-	
No	241 (75.4)	3.6 ± 1.1	-	

A total of 320 children were included in the analysis. The association between family characteristics and average screen time is presented in Table 3.

As shown in Table 3, significant variations in children’s screen time were observed across several sociodemographic factors. Children from nuclear families had higher mean screen time compared to those from joint families (4.5 ± 1.2 versus 3.2 ± 1.0 hours/day; t = 7.82, p < 0.001). Similarly, children of illiterate mothers demonstrated significantly higher screen exposure than those of literate mothers (4.1 ± 1.2 versus 3.4 ± 1.1 hours/day; t = 2.94, p = 0.004).

Although children of illiterate fathers had a higher mean screen time compared to those of literate fathers (4.6 ± 1.3 versus 3.7 ± 1.1 hours/day), this difference was not statistically significant (t = 1.32, p = 0.187). A significant association was observed with maternal employment, with higher screen exposure among children of employed mothers compared to unemployed mothers (4.6 ± 1.3 versus 3.5 ± 1.1 hours/day; t = 5.48, p = 0.002).

In contrast, no statistically significant association was found with paternal employment status, although higher mean screen time was observed among children of unemployed fathers (4.6 ± 1.0 versus 3.2 ± 1.2 hours/day; t = 0.76, p = 0.382). Notably, the highest screen exposure was observed among children from households where both parents were working (4.8 ± 1.3 versus 3.6 ± 1.1 hours/day; t = 6.21, p = 0.001).

These observations indicate that family structure, maternal education, and maternal employment, particularly in households where both parents are working, play an important role in determining children’s daily screen exposure.

Higher screen time was observed among children of working mothers and was most pronounced in dual-income families. In contrast, children from joint family settings demonstrated comparatively lower screen exposure. These findings suggest that variations in parental availability and caregiving structure may influence children’s engagement with screens (Table 3).

The present study evaluated the contextual use of screen-based devices as a “digital pacifier” among caregivers. During feeding, a considerable proportion reported frequent usage, with 132 (41.3%) indicating “often” and 58 (18.1%) “always” providing screens, whereas 98 (30.6%) reported occasional use and 32 (10.0%) reported never using screens in this context. Similarly, during household activities, 111 (34.7%) caregivers reported “often” and 64 (20.0%) “always” using screen exposure, reflecting its common use as a strategy to manage children while attending to routine tasks.

Screen use was especially evident in situations requiring behavioral management. To calm crying, 138 (43.1%) caregivers reported “often” and 104 (32.5%) “always” using screens, with only 16 (5.0%) indicating that they never relied on them. In a similar pattern, during travel and when attempting to keep the child quiet, 124 (38.8%) caregivers reported “often” and 96 (30.0%) “always” using screens. In public settings, 110 (34.4%) reported “often” and 84 (26.3%) “always” using screen exposure to maintain calm behavior or silence.

**Figure 2:**
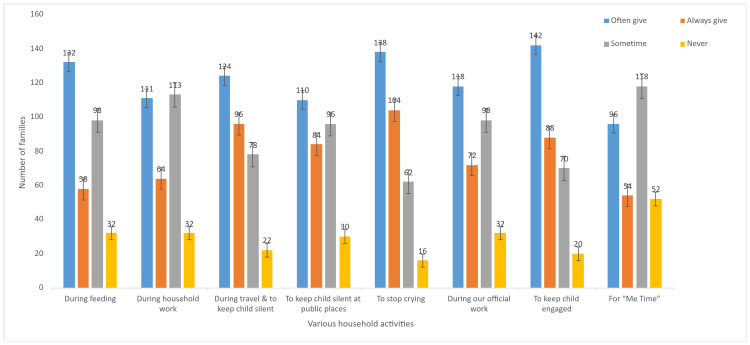
Context-wise distribution of screen use (“digital pacifier”) practices among caregivers (N = 320)

During work-related tasks, 116 (36.3%) caregivers reported “often” and 72 (22.5%) “always” using screens, while 100 (31.3%) indicated occasional use. For general engagement, the highest proportion of “often” use was observed, with 140 (43.8%) caregivers frequently using screens, followed by 88 (27.5%) reporting “always” use. Additionally, for personal leisure or “me time,” 94 (29.4%) caregivers reported “often” and 54 (16.9%) “always” using screens (Figure 2).

Overall, these findings demonstrate that the use of screens as a “digital pacifier” is highly prevalent across a range of daily situations, particularly for behavioral regulation and caregiver convenience, with comparatively fewer caregivers reporting complete avoidance across most contexts.

## Discussion

The findings of this study should be interpreted as associations rather than causal relationships. Our observational findings indicate that digital media use is highly prevalent among Indian children aged 1-5 years, with a considerable proportion surpassing recommended daily exposure limits. Digital platforms, including smartphones, tablets, and televisions, are frequently employed as “digital pacifiers” by parents and grandparents to soothe, distract, or engage children. Although this approach offers immediate convenience, our observations suggest that excessive reliance may be linked to decreased physical activity, disrupted sleep patterns, and early indications of delays in language, cognitive, and social development. These findings reflect a broader shift toward high levels of digital engagement during early childhood across Indian settings.

Shirley et al. demonstrated that increased screen exposure among children in Tamil Nadu was associated with reduced participation in physical play and greater sedentary behavior [8]. Consistent with this, our study observed that frequent use of digital devices as calming tools often substitutes time otherwise spent in creative or exploratory activities. This trend is concerning, as active play during early childhood is essential for motor development, problem-solving abilities, and imaginative growth.

Krupa et al. reported that excessive screen use negatively affected reciprocal mother-child interactions, particularly among children with autism spectrum disorders [9]. Similarly, our findings suggest that overdependence on digital pacifiers may reduce both the frequency and quality of interactions between children and caregivers, including parents and grandparents. Such reduced engagement can limit opportunities for social learning, emotional bonding, and responsive communication, all of which are fundamental for language acquisition and socio-emotional development.

John et al. identified an association between higher screen exposure and parent-reported cognitive delays in preschool children in Kerala, particularly affecting attention and language domains [10]. Our observations are in agreement, as children with greater daily screen exposure often demonstrate delayed language milestones and reduced participation in interactive or problem-solving activities. Additionally, Pasi et al. reported a marked increase in children’s screen time during the COVID-19 pandemic, largely attributed to lockdown-related restrictions, which also contributed to reduced physical activity and altered sleep patterns [11]. Comparable trends were observed in our study, where reliance on digital pacifiers appeared to increase during periods of limited outdoor activity and social interaction.

Gandhi et al. [12] and Varadarajan et al. [13] have shown that screen-based media exposure is associated with lower levels of physical activity and a higher prevalence of developmental concerns across cognitive, motor, and social domains. These observations align with our findings, where children frequently exposed to digital devices as pacifiers had fewer opportunities for outdoor play and imaginative engagement, potentially restricting holistic development.

Kaur et al. [14] and Joseph et al. [15] highlighted the negative impact of excessive screen exposure on sleep, particularly when devices are used during evening hours or as soothing tools before bedtime. In our study, many caregivers reported using digital devices to calm children prior to naps or nighttime sleep, which may inadvertently contribute to delayed sleep onset and irregular sleep cycles. These patterns underscore the importance of promoting mindful and structured use of digital media rather than habitual reliance for behavioral management.

Agrawal et al. [16] and Gayathri et al. [17] identified socioeconomic status, parental education, and access to digital devices as key determinants of screen exposure. In our observations, households with easier access to digital devices demonstrated greater reliance on digital pacifiers, with children in these settings more likely to experience prolonged screen use. These children also exhibited reduced engagement in active play, delayed language progression, and lower levels of social interaction, reinforcing the influence of environmental and familial factors on early digital habits.

Within household settings, multiple situational factors contribute to the use of mobile devices among young children. Kulakci-Altintas reported that managing challenging behavior is a primary reason caregivers introduce mobile devices [18]. Devices are often used to stop crying, reduce irritability, or manage tantrums, particularly when caregivers are constrained by time or competing demands. The immediate and immersive nature of digital content enables rapid behavioral control, reinforcing repeated use. Over time, such patterns may condition children to associate screens with emotional regulation, gradually replacing traditional soothing strategies.

Shah and Phadke further noted that caregivers frequently rely on mobile devices in social and public environments [19]. Devices are commonly used during family gatherings, religious events, dining situations, and travel to maintain calm behavior. While caregivers often perceive this as a practical approach to avoid disruption, repeated use in such contexts may normalize dependence on screens beyond the home environment.

Collectively, these findings suggest that although digital devices provide short-term relief and convenience, frequent reliance on them as digital pacifiers may carry unintended developmental risks. Our study offers real-world evidence that reducing excessive screen exposure and encouraging alternative activities, such as active play, storytelling, and meaningful caregiver-child interaction, can promote healthier developmental outcomes. Interventions focusing on caregiver awareness, practical behavioral strategies, and culturally appropriate guidance are essential to ensure that digital media is used in a balanced manner without compromising physical, cognitive, or socio-emotional development.

Limitations

This study has several limitations. First, its cross-sectional design allows identification of associations but precludes causal inferences. Second, reliance on caregiver-reported data may introduce recall and social desirability bias, potentially affecting the accuracy of reported screen use and behavioral patterns. Third, the questionnaire, although structured and domain-based, did not undergo formal psychometric validation, which may influence the reliability and generalizability of findings. Fourth, the study did not assess the nature or quality of digital content, which may differentially impact developmental outcomes. Fifth, the study population, although reasonably representative, may not fully capture the diversity of cultural and socioeconomic contexts across India, limiting generalizability. Sixth, autism spectrum disorders and other developmental conditions were not specifically assessed, and differential access to diagnostic services may introduce unmeasured confounding or selection bias. Seventh, gender-based differences in screen exposure were observed, but underlying sociocultural determinants were not explored, which may contribute to residual confounding. Finally, other potential confounders, including parental screen use, parenting practices, home media environment, availability of play spaces, and sleep routines, were not comprehensively evaluated and may have influenced the observed associations.

## Conclusions

The present study demonstrates that digital media exposure is highly prevalent among Indian children aged 2-5 years, with a substantial proportion exceeding recommended usage limits. Parents and grandparents commonly rely on digital devices as “digital pacifiers” to manage children’s behavior, often without full awareness of the potential developmental implications. Higher levels of screen exposure were associated with reduced participation in active play, disrupted sleep patterns, and early indicators of delays in language, cognitive, and social domains. These findings suggest that habitual reliance on digital devices for behavioral management may influence early childhood development.

Although causal relationships cannot be established within the scope of this observational study, the patterns identified highlight the need to limit excessive dependence on digital pacifiers and to encourage developmentally appropriate alternatives. Promoting activities such as active play, reading, and interactive engagement between children and caregivers, including parents and grandparents, may help mitigate the adverse effects of prolonged screen exposure. The development of culturally relevant guidelines and practical strategies for caregivers is essential to ensure that digital media serves as a supportive tool for learning and recreation without undermining healthy physical, cognitive, and socio-emotional growth. Thoughtful and balanced use of digital devices can enable children to benefit from technology while preserving opportunities for creativity, exploration, and meaningful interpersonal interaction.

## Appendices

Table 4 presents the detailed questionnaire used in the present study.

**Table 4:** Questionnaire: situation-based use of mobile phone as digital pacifier Instruction: How often do you give a mobile phone to your child in the following situations? (Tick one option for each.)

Situation	Item number	Statement	Never	Sometimes	Often	Always
1. During Feeding	1.1	Do you give your child a mobile phone during feeding?	☐	☐	☐	☐
	1.2	Does feeding become easier when your child is using a mobile phone?	☐	☐	☐	☐
	1.3	Does your child expect a mobile phone during feeding?	☐	☐	☐	☐
2. During Household Work	2.1	Do you give your child a mobile phone while doing household work?	☐	☐	☐	☐
	2.2	Do you give a phone to keep your child occupied during chores?	☐	☐	☐	☐
	2.3	Is it difficult to complete household work without giving a phone?	☐	☐	☐	☐
3. During Travel	3.1	Do you give your child a mobile phone during travel?	☐	☐	☐	☐
	3.2	Do you use a phone to prevent your child from becoming restless during travel?	☐	☐	☐	☐
	3.3	Does your child demand a phone during travel?	☐	☐	☐	☐
4. In Public Places	4.1	Do you give your child a mobile phone in public places?	☐	☐	☐	☐
	4.2	Do you use a phone to avoid disturbance in public settings?	☐	☐	☐	☐
	4.3	Do you feel pressure to keep your child quiet using a phone in public?	☐	☐	☐	☐
5. To Stop Crying	5.1	Do you give a mobile phone when your child cries?	☐	☐	☐	☐
	5.2	Does a mobile phone help stop your child’s crying quickly?	☐	☐	☐	☐
	5.3	Do you depend on a phone to calm your child?	☐	☐	☐	☐
6. During Work	6.1	Do you give your child a mobile phone while you are working?	☐	☐	☐	☐
	6.2	Do you use a phone to avoid interruption during work?	☐	☐	☐	☐
	6.3	Are you unable to complete your work without giving a phone?	☐	☐	☐	☐
7. To Keep Child Engaged	7.1	Do you give a mobile phone to keep your child engaged?	☐	☐	☐	☐
	7.2	Does your child prefer the phone over other activities?	☐	☐	☐	☐
	7.3	Do you use a phone when no other activity is available?	☐	☐	☐	☐
8. For “Me Time”	8.1	Do you give a mobile phone to get personal time?	☐	☐	☐	☐
	8.2	Do you use a phone when you feel tired or need rest?	☐	☐	☐	☐
	8.3	Do you find it difficult to get personal time without giving a phone?	☐	☐	☐	☐
